# Periorbital Ecchymosis and Subconjunctival Hemorrhage following Ear Surgery

**DOI:** 10.1155/2013/791068

**Published:** 2013-09-30

**Authors:** Mohsen Rajati, Mehdi Bakhshaee, Kamran Khazaeni

**Affiliations:** Sinus and Endoscopic Surgery Research Center, Qaem Hospital, Faculty of Medicine, Mashhad University of Medical Sciences, Mashhad 9176699199, Iran

## Abstract

*Objective*. To evaluate the occurrence of two periorbital complications of surgery for Chronic Suppurative Otitis Media (CSOM) and discuss the potential pathophysiologic mechanisms. *Materials and Methods*. This is a retrospective review of the CSOM surgeries performed between Oct, 2005, and Jan, 2011, in our hospital. The early postoperative conditions of the patients were scrutinized to identify periorbital ecchymosis and subconjunctival hemorrhage. *Results*. Eight cases out of 756 patients were noted to have periorbital ecchymosis, and two of the patients also had simultaneous subconjunctival hemorrhage. All cases in which the complications occurred had undergone tympanoplasty, and in three patients mastoidectomy had also been performed. The age of the affected patients ranged from 24 to 70 years old. In all of them the condition ensued the day after the surgery and became better within 5 to 10 days. Complete recovery took approximately 1 month. *Conclusion*. Periorbital ecchymosis and subconjunctival hemorrhage are rare but safe complications of ear surgeries. The conditions are self-limiting and no management is necessary.

## 1. Introduction

Chronic Suppurative Otitis Media (CSOM) is a fairly common disease, and it is usually managed with surgery. In most otology wards the most common surgical procedures are those performed on patients with CSOM and, like any other medical intervention, complications can happen. These complications include those related to anesthesia, as well as otologic and intracranial complications. Problems related to the skin and wound healing such as infection, hematoma, and dehiscence are considered minor complications in CSOM surgeries. There have been various case reports on rare, out of the ordinary mishaps, in these surgeries. Here, we describe periorbital ecchymosis with or without subconjunctival hemorrhage as a rather rare complication of ear surgeries. Although a familiar condition in nose and sinus operations to the extent of our knowledge, periorbital problems in otologic surgeries were first brought up by Rudnick et al. who described 4 cases of periorbital edema and cellulitis in 97 patients with cochlear implants [1]. Herein, we discuss periorbital ecchymosis, which is a more complex condition compared to edema and/or erythema of the orbital area. 

## 2. Materials and Methods 

This is a retrospective review of the occurrence of periorbital ecchymosis and/or subconjunctival hemorrhage as a complication following ear surgeries including tympanoplasty with or without mastoidectomy in our university hospital, which is a tertiary referral medical center. Intravenous cephalosporin antibiotics were given prophylactically to all patients just before surgery and then given orally after surgery for an additional 5 days. The postoperation protocol at our centre is as follows: a compressive bandage is applied for all cases in the operation room and a first reevaluation is performed on the day after surgery when the bandage is changed and the next visits are on the third and seventh day at the time of removal of the bandage and sutures, respectively. Then periodic monthly examinations are performed for up to 3 months. The follow-up visits are arranged according to the patient's condition. The surgeries evaluated here were performed between Oct, 2005, and Jan, 2011. Cases with the mentioned ocular complications were taken into consideration, and the clinical features were reviewed. The intensity of edema, periorbital ecchymosis, and subconjunctival hemorrhage was determined visually according to the authors' classification (Table 1). All cases were followed at least for one year.

## 3. Results

**Figure 1:**
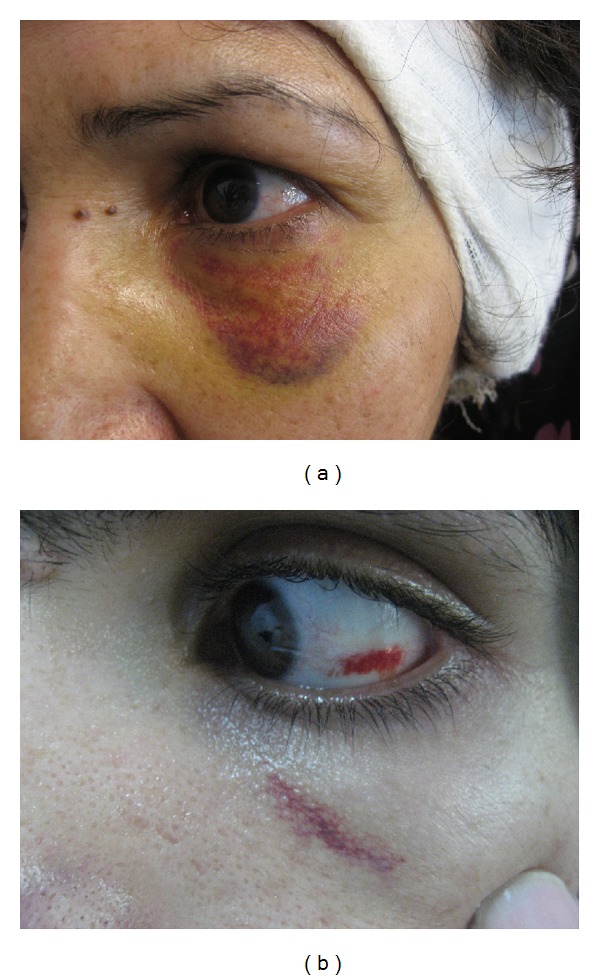
Two cases with severe periorbital ecchymosis (a) with mild periorbital ecchymosis and with subconjunctival hemorrhage (b) after tympanoplasty.

**Figure 2:**
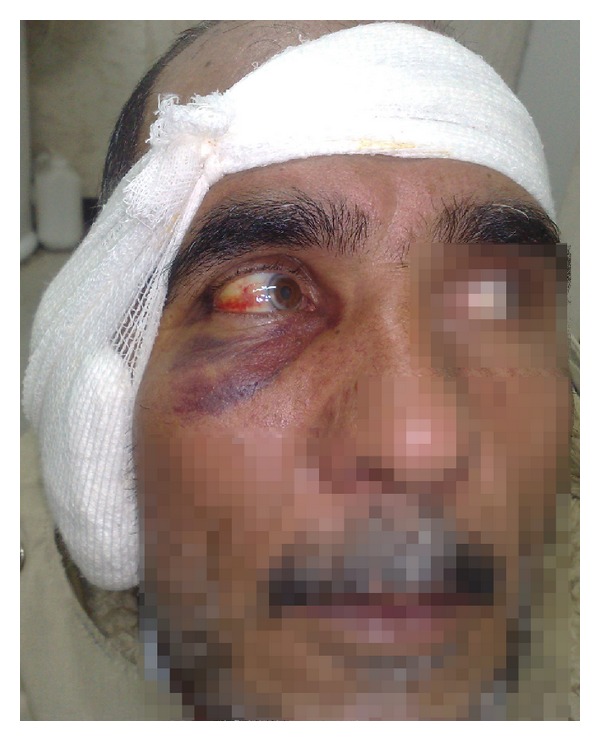
A case with severe periorbital ecchymosis and mild subconjunctival hemorrhage after tympanomastoidectomy.

The total number of patients included in the study was 756, their mean age was 31.3 ± 12.0 years (min: 6; max: 72). Of the patients, 468 (61.9%) and 288 (38.1%) were female and male, respectively. The types of operations the patients underwent were as follows: tympanoplasty (412 cases), tympanoplasty with mastoidectomy (244 cases), modified radical mastoidectomy (63 cases), and ossiculoplasty (37 cases). Eight cases were noted to have periorbital ecchymosis (Figures 1 and 2); two of them had simultaneous subconjunctival hemorrhage as well (Figures 1(b) and 2). The ages of these patients ranged from 24 to 70, and the patients' data are shown in Table 2. 

In all cases the first presentation of the complications was on the day after surgery *not* in the operating room and the conditions worsened within 2 to 3 days. None of the patients suffered from disturbances in visual acuity although mild to moderate edema was an accompanying feature of the ecchymosis. It took around 5 to 10 days for the ecchymosis to be partially resorbed, but complete resolution of edema and discoloration mostly occurred within 1 month. None of the patients complained from pain, tenderness, itching, tearing, or limitation of ocular motility. No systemic symptoms, such as fever, chill, fatigue, headache, or rhinorrhea were observed.

## 4. Discussion

Periorbital edema and ecchymosis have several etiologies such as fractures of the base of the skull (also called raccoon eye), fractures of the facial bones, rhinoplasty, endoscopic sinus surgery, sinusitis, superficial eyelid cellulitis, preseptal infections, and allergic reactions. In these conditions different mechanisms have been proposed to describe how the edema or ecchymosis is induced. Most of these mechanisms pivot around the vascular, lymphatic, and soft tissue anatomy of the periorbital area. There are also various explanations for blood accumulation around the eye following ear surgeries, some of which will be covered below.

The skin of the eyelids is not thick, and there is only a thin layer of connective tissue between the skin and the underlying muscular layer. This lean and loose layer of connective tissue is where accumulation of fluid, such as blood, happens following injuries [2]. The arterial supply to the eyelids originates from several vessels including the supratrochlear, supraorbital, lacrimal, and dorsal nasal arteries (from the ophthalmic artery); the angular artery (from the facial artery); the transverse facial artery (from the superficial temporal artery); and branches from the superficial temporal artery itself. The temporal and infratemporal area on the other hand are nourished by terminal branches of the external carotid artery. The superficial temporal artery branches in the temporoparietal fascia and supplies the skin and soft tissue in the temporal area. The internal maxillary artery also has anterior and posterior temporal branches which travel on the deep surface of the temporal muscle and contribute blood to the periorbital area [2].

In addition to the eyelids and the blood supply to the region, the fascia layers and planes in the area between ear and eye deserve consideration as to their role in the development of periorbital ecchymosis. There are three basic layers which are a continuation of each other: the Superficial Muscular Aponeurotic System (SMAS, in the face), the galea (in the forehead); and the temporoparietal fascia (in the temple). The SMAS acts as a carrier for superficial fat layers in the midface, where true and false retaining ligaments create a complex network connecting the dermis to the fascia layers through the superficial fat. In the temple, the superficial fat is sparser and there are no retaining ligaments [3], so in this plane the resistance to either blood or hydrostatic pressure changes is decreased, contrary to the midface where there is dense superficial fat and stronger adhesions.

Rudnick et al. reported 4 cases of periorbital edema and preseptal cellulitis in 97 pediatric cochlear implantees [1]. As orbital complications of sinusitis are far more common in children [4], the researchers believed preoperative rhinosinusitis was the most important predisposing factor for edema and cellulitis. Hoffman et al. challenged this notion, maintaining that large skin flaps were the most prominent issue and that the process is *not* infectious and is self-limiting [5]. Aside from attempting to understand what the mechanism of edema is, there were no cases of ecchymosis in the report by Rudnick et al. As mentioned before the 8 cases in this report had ecchymosis with or without edema. The number of cases included here would have been dramatically increased if cases with only edema had been included. Another factor that differs between this study and the study by Rudnick et al. is that in cochlear implant surgery; there is no need to harvest a temporalis fascia graft. In our center we also reviewed the records of 375 cochlear implants and found *no one* with periorbital ecchymosis.

In all of the patients who developed ecchymosis the surgical approach was postauricular, and a graft was harvested from the temporalis fascia. One possibility is that injury to some branches of the superficial temporal vein caused disorder in the venous drainage of the periorbital area and that the elevated hydrostatic pressure resulted in extravasation of red blood cells and ecchymosis formation. Individual anatomic variation of the vascular structure in the periauricular and periorbital area would account for the rarity of the condition. Graft harvesting was the one common event in these 8 patients, and mastoid drilling was done in only 3 of them. None of these patients had systemic hypertension or were taking anticoagulants or acetylsalicylic acid.

Various external factors that are not central to the surgical procedure may also play a role in the development of ecchymosis. However, while a tight bandage and dressing may have a role in the development of edema, unilateral ecchymosis seems unlikely to be caused by such circular pressure around the skull. The presumption of direct trauma to the area is also not acceptable. Vigorous coughing during extubation is another possible source of subconjunctival hemorrhage but the likelihood of it happening on the same side as the surgery and with concomitant ecchymosis in the periorbital skin makes it unlikely in the present subjects. Several otologists do some elaborate soft tissue manipulation in the area just above the external ear canal which is the root of zygoma and believe that it is necessary for a better exposure to the middle ear or for mastoid drilling. Going too far anteriorly in the zygoma root may endanger the vessels closer to the orbit. 

In contrast to ear surgery, periorbital edema and ecchymosis are well known in rhinoplasty. Considering the location of the osteotomies there is a high risk of damage to the angular vessels or their small branches, and this probably explains the mechanism of periorbital changes during rhinoplasty [6]. Techniques are also constantly being introduced to reduce the intensity and/or duration of these symptoms such as using steroids, local injection of a combination of lidocaine and adrenaline, and creating a subperiosteal tunnel [7–11]. However, in ear operations the field of surgery is by no means as close to the ecchymotic area as in rhinoplasty. Interestingly though, in all cases skin discoloration happened in the day after the surgery not immediately on the operation table. The same thing happens in rhinoplasty-associated ecchymosis.

One last point to consider is the adverse effects of anesthesia. It seems unlikely that anesthesia plays a role in periorbital changes following ear surgeries. Some symptoms such as bilateral blurred vision have been proposed which seem to be related to the anticholinergic side effect of some anesthetic agents. Corneal abrasion as a result of careless eye coverage may also happen more frequently in head and neck surgeries [12, 13]. But our subject of discussion is a totally different point. Another alternative matter is the use of a local injection of adrenaline solution in the postauricular area. Kumar and Moturi reported a case of subconjunctival hemorrhage following extraction of maxillary first and second molar teeth and postulated that the injection of the anesthetic solution may have had a role either by injuring the deep vessels in the pterygomaxillary and infratemporal spaces or by untoward spread of the solution to ectopic sites (i.e., the periorbital area) causing peculiar ocular symptoms [14]. Considering the distance between the injection site and the deep facial vessels, we believe this theory can *hardly* explain our cases.

## 5. Conclusion

Periorbital edema and ecchymosis with accompanying subconjunctival hemorrhage are rare complications of tympanoplasty or mastoid surgeries. Although they may be worrisome to the patients and their families, these are self-limiting and essentially none dangerous conditions. As an otologist being aware of the potential for the development of these complications prevents redundant diagnostic or therapeutic measures. 

## Figures and Tables

**Table 1:** The classification of the severity of orbital involvement.

	Mild	Moderate	Severe
Edema	Normal palpebral fissure	Narrowed palpebral Fissure	Closed palpebral fissure
Periorbital Ecchymosis	Less than half of palpebral involvement	Half of palpebral involvement	Total palpebral involvement
Subconjunctival Hemorrhage	Less than half of conjunctival involvement	Half of conjunctival involvement	Total conjunctival involvement

**Table 2:** Demographic characteristic of the patients with priorbital ecchymosis and subconjunctival hemorrhage.

Cases	Age	Type of surgery	Edema	Periorbital ecchymosis	Subconjunctival hemorrhage
1	27	Revision tympanoplasty with mastoidectomy	Mild	Severe	None
2	24	Tympanoplasty	None	Mild	Mild
3	54	Tympanoplasty with mastoidectomy	Moderate	Moderate	None
4	32	Tympanoplasty	Moderate	Mild	None
5	26	Tympanoplasty	Moderate	Mild	None
6	70	Tympanoplasty	Moderate	Severe	Moderate
7	48	Tympanoplasty with mastoidectomy	Mild	Severe	None
8	57	Tympanoplasty	Mild	Severe	None
